# Communication strategies used by medical physicians when delivering bad news at the Maputo Central Hospital, Mozambique: a cross-sectional study

**DOI:** 10.1186/s12904-023-01309-y

**Published:** 2023-11-22

**Authors:** Natália Ubisse Schmauch, Emilia Pinto, Francisca Rego, Luísa Castro, Jahit Sacarlal, Guilhermina Rego

**Affiliations:** 1 Faculty of Medicine, University of Porto, Porto, Portugal; 2 Pain Unit, Maputo Central Hospital, Maputo, Mozambique; 3 Center for Research in Health Technologies and Services - CINTESIS, University of Porto, Porto, Portugal; 4 Faculty of Medicine, Eduardo Mondlane University, Maputo, Mozambique

**Keywords:** Health Communication, Education, Medical, Palliative care

## Abstract

**Background:**

Physicians’ communication with patients and their families is important during both the disease diagnosis and prognosis stages and through the follow-up process. Effective physician communication improves patients’ quality of life and satisfaction with care and helps reduce suffering for those newly diagnosed with advanced progressive illnesses. This study aims to identify the communication strategies physicians use in the transition to palliative care and how these professionals perceive their academic and clinical preparation concerning this task.

**Methods:**

A cross-sectional and quantitative study. Physicians providing palliative care at the Maputo Central Hospital, Mozambique, were invited to complete a 17-question questionnaire. This questionnaire was based on a Brazilian adaptation of the Setting-Perception-Invitation-Knowledge-Emotions-Strategy (SPIKES) tool, the P-A-C-I-E-N-T-E protocol, with additional questions regarding socio-demographic details and the integration of “communication of bad news” into hospital training.

**Results:**

Of the 121 participants, 62 (51.2%) were male, and 110 (90.9%) were general practitioners, with a median age of 36 years old. They had worked in clinical practice for a median of 8 years and in their current department for three years. The majority of the participants considered that they have an acceptable or good level of bad news communication skills and believed that they do it in a clear and empathic way, paying attention to the patient’s requests and doubts; however, most were not aware of the existing tools to assist them in this task and suggested that delivering bad news ought to be integrated into the undergraduate medical course and included in hospital training.

**Conclusions:**

This study adds to our understanding of physicians’ strategies when communicating bad news in the context of palliative care at one Mozambique hospital. As palliative care is not fully implemented in Mozambique, it is important to use protocols suitable to the country’s healthcare level to improve how doctors deal with patients and their family members.

## Background

Health communication is a dynamic and transversal process that establishes a bond between the patient, the family, the multidisciplinary team, and the institution [1]. **‘**Bad news’ can be defined as any information that negatively changes an individual’s expectations about their present and future [2]. Communication is an act of great complexity. From a humanitarian perspective, the quality of communication determines the development of society, scientific improvements, and the level of civility. In worst-case scenarios, poor communication contributes to tension, stagnation, and regression [3].

According to the World Health Organization (WHO), palliative care is an approach delivered by a multidisciplinary team to improve the quality of life of patients and their relatives dealing with a life-threatening disease [4]. Early identification, correct assessment, and management of pain and other symptoms (either physical, psychological, social, or spiritual) can help to prevent and relieve suffering [4].

Communication is an indispensable and therapeutic element in the context of palliative care, due to its power to improve the quality of life, preserving the autonomy of patients and respective families. Initial ‘bad news’ communication is a physician’s responsibility to explain the patient’s condition and the available therapeutic options, provide information about the prognosis, and answer any questions or concerns [5]. However, breaking bad news is not easy, even for those who face it daily, such as healthcare professionals. It is more challenging when culture, religious beliefs, and socioeconomic factors influence how patients, and their families deal with bad news [6]. One potential solution is to train newly graduated physicians to prepare them for the different situations they may encounter when communicating bad news in the context of palliative care [7]. The quest to build the physician’s capabilities to break bad news is not new, with numerous mnemonics, like SPIKES [8], NURSE [9] and I PREPARE [10], and teaching approaches available [11]. Various validated questionnaires are available and are applied to assess physician’s knowledge and attitudes toward delivering bad news [12–15].

Buckman’s (1992) Setting-Perception-Invitation-Knowledge-Emotions-Strategy (SPIKES) protocol is one of the best-known tools (mnemonic) used to assist in the process of breaking bad news [8]. It includes four essential objectives for communicating bad news: collecting information about the patient, transmitting medical information, providing support to the patient, and inviting their input into determining the treatment strategy or plan [8, 16]. More recently, the P-A-C-I-E-N-T-E protocol, adapted from SPIKES, has been available to Brazilian Physicians to guide breaking bad news conversations with patients with advanced progressive illness [5].

Studies undertaken in countries with different socio-economic circumstances, cultures, and access to healthcare, such as Iran, the United States of America (USA), and Brazil, demonstrated that most physicians lack the training to communicate bad news effectively [17–21]. Furthermore, physicians reported that they did not receive enough training during undergraduate courses and were unfamiliar with SPIKES protocol or any other instrument to assist with this task; rather developed their communication skills by observing their more experienced colleagues [20, 21]. These studies demonstrated that physicians with more experience were more comfortable delivering bad news [17, 18]. However, more experienced physicians were unaware of the SPIKES protocol, which is concerning, since younger physicians see them as professional role models [20].

This issue is especially important in low-income countries like Africa since palliative care has only recently been introduced [22], and its routine inclusion in the health systems is precarious [23]. There are very few African studies reporting on palliative care communication. A South African qualitative study exploring physicians’ communication skills when transmitting a bad prognosis to the patient and family members found that those who dealt better with this situation had greater palliative care knowledge and worked as part of a multidisciplinary team [24]. The authors concluded that it is important for the Physician to have a support team, including a psychologist [24]. Regardless of their expertise, even with developed communication skills and knowledge about protocols for communicating bad news, physicians may not be able to cope with individuals’ diverse needs and emotions in the transition to palliative care [24].

### Context

In 2001, pain consultation began in Maputo Central Hospital (MCH), Mozambique, leading to the opening of an independent Pain Unit in 2007, becoming a national pain relief reference. Given the increase in oncologic patients, a palliative care service was implemented in this unit in 2019 with multidisciplinary teams involved. Patients from the different hospital services were referred to palliative care in inpatient or outpatient care [25]. However, there is still a shortage in the provision of palliative care in Mozambique, despite the daily contact of physicians with palliative patients. The care offered to the patients does not have a holistic perspective, focusing mainly on the patient’s treatment but lacking spiritual care and family support [26]. This reflects a lack of physician knowledge about the difficulties of communicating bad news [26]. In MCH, palliative care is available to patients with advanced cancer and diverse severe non-oncologic diseases. Most patients with advanced disease admitted to MCH have been diagnosed late, are seriously ill, and have little understanding of their disease or palliative care [27]. Despite Mozambique’s progress in developing palliative care services, a lack of funding, resources, and availability of medicines limits its availability [25]. While palliative care content has been included in undergraduate health professionals’ curricula, there is a lack of continuous training during medical practice [26].

This study aims to identify the communication strategies physicians use in the transition to palliative care and how these professionals perceive their academic and clinical preparation concerning this task.

## Methods

### Design, study setting, and participants

A cross-sectional and quantitative study reported following the STROBE guidelines [28]. The Maputo Central Hospital (MCH), Mozambique, Medicine, Surgery, Gynecology and Obstetrics, Orthopedics inpatient and outpatients Departments where doctors care for patients with palliative needs is the setting for this study, undertaken between September 2 and November 29 of 2019.

### Survey

The survey included a socio-demographic section (age, sex, clinical practice years, professional category, and service) and the P-A-C-I-E-N-T-E questionnaire with 17 Likert, Yes/No, or multiple-choice questions [5]. This questionnaire was designed to assess physicians’ knowledge and understanding of delivering bad news and determine how prepared they feel to complete this task. The questions were organized into five sections: (1) sociodemographic; (2) bad news definition, frequency of communication of bad news, and the way they are given (Q1-Q11 and Q14); (3) physicians’ feelings when breaking bad news (Q12-Q13); (4) how they learned to deliver bad news; and (5) knowledge of other communication instruments or resources to assist with breaking bad news (Q15-Q17).

The face and content validity of the P-A-C-I-E-N-T-E tool was assessed by an expert committee (the authors of the manuscript) with palliative care expertise and experience in communicating bad news to patients and their families. Each of these experts independently reviewed the relevance, clarity, and comprehensiveness of the questions and considered whether they adequately covered the intended characteristics of the concept. An extra closed-ended question was added to capture participants’ views about the need to include communicating bad news in physicians’ hospital training (Q18). The final questionnaire (including the additional question) was administered to a convenience sample of physicians (n = 10) not working in Departments caring for palliative patients, with no further changes to the questionnaire deemed necessary.

### Sample and eligibility criteria

Doctors employed in the identified departments during the study period for more than three months were eligible to participate.

All eligible doctors were invited to complete a self-filling paper questionnaire once they had signed the Informed Consent Form.

### Statistical analysis

Data was extracted from the paper questionnaires and entered into a Microsoft Excel® 2016 (USA) spreadsheet. Statistical analyses were performed using IBM SPSS® Statistics (version 26.0; SPSS Inc., Chicago, Illinois, USA). Categorical variables were described by absolute and relative frequencies. The normality of quantitative variables, age and time of practice, was verified by visual inspection of histograms and confirmed with the Kolmogorov-Smirnov normality test. Since all deviated from normality, quantitative variables were described by the median and interquartile interval [1st Q; 3rd Q], and correlation between variables was computed with the Spearman correlation coefficient [29]. The comparison of categorical variables between groups was performed using the chi-square test or the Fisher’s exact test (when more than 20% of the expected counts were under 5 in a 2 × 2 contingency table; for larger tables, the Fisher-Freeman-Halton exact test was used) [29]. For the comparison of ordinal or quantitative variables between two groups, the non-parametric Man-Whitney test was used [29]. Given the exploratory nature of our study, no Bonferroni correction for multiple testing was applied [30]. P-values were considered significant if less or equal to 0.05.

### Ethics

The study was reviewed, approved, and authorized by the Institutional Committee on Bioethics in Health of the Faculty of Medicine/Maputo Central Hospital (CIBSFM & HCM/84/2018) and by the Scientific and Pedagogical Directorate of Maputo Central Hospital (531/DCIEFP/HCM/19).

## Results

Among the 121 participants, half (n = 62, 51.2%) were male, and the median age [1st Q; 3rd Q] was 36 [32; 41.8] years. The median length of time in practice was 8 [5; 13.3] years overall and 3 [1; 7] years in the current department.

The majority (90.9%) were general practitioners with a smaller number of specialist physicians (n = 11, 9.1%), with almost two-thirds working in medicine, with the remainder working in surgery (17.4%); Gynecology and Obstetrics (14%), or Orthopedics (5.8%, see Table 1 for details).

**Table 1:** Physicians’ demographic and professional characteristics (N = 121)

Variables	Descriptives
Age in years^a^, med [1st Q; 3rd Q]	36 [32; 41.8]
Practice years^b^, med [1st Q; 3rd Q]	8 [5;13.3]
Practice years in actual service^c^, med [1st Q; 3rd Q]	3 [1; 7]
Sex, n (%)	
Male	62 (51.2)
Female	59 (48.8)
Professional category, n (%)	
General Clinic	110 (90.9)
Specialist	11 (9.1)
Services, n (%)	
Gynecology/Obstetrics	17 (14)
Medicine	76 (62.8)
Surgery	21 (17.4)
Orthopedics	7 (5.8)

^a, b, c^ Only 120, 118 and 119 professionals gave information, respectively.

### Bad news definition, forms of communication, and frequency

Most participants (95.9%) correctly identified bad news (Q1) and a high percentage of them considered their ability to deliver bad news as acceptable (52.1%) or good (30.6%) (Q4) (Table 2).

**Table 2:** Bad news: definition, frequency and skills of physicians in their communication (N = 121)

Questions	n (%)
**1) What is bad news?**	
Only delivering bad news of death	1 (0.8)
Any information that implies negative change and would affect the individual’s vision on life	116 (95.9)
All information that results in physical damage to the patient	4 (3.3)
**2) How often do you deliver bad news?**	
Never	1 (0.8)
Very Seldom	19 (15.7)
Occasionally	50 (41.3)
Frequently	30 (24.8)
Almost always	21 (17.4)
**3) How do you deliver bad news?**	
Verbally only	45 (37.2)
Verbally and non-verbally (touching, looking, with empathy…)	76 (62.8)
**4) How do you rate your ability to deliver bad news?**	
Bad	2 (1.7)
Acceptable	63 (52.1)
Good	37 (30.6)
Very Good	19 (15.7)
**5) Where do you deliver bad news?**	
I look for a private and comfortable place	74 (61.2)
I informally report in the corridor or elsewhere outside the office	2 (1.7)
I break the news in an available office	45 (37.2)
**6) How do you position yourself when delivering bad news to a bedridden patient?**	
I inform the patient, whilst standing beside the bed when the patient is bedridden	74 (61.2)
I inform the patient whilst sitting alongside the bed when the patient is bedridden	47 (38.8)
^+^ **7) How do you provide bad news?**	
With clear, understandable language, avoiding technical words	110 (90.9)
I explain everything in detail	25 (20.7)
I provide expectance of hope even though there is none	7 (5.8)
I establish a relationship of trust	34 (28.1)
I explain in a detailed and technical way	2 (1.7)
I put myself in the patient’s shoes	42 (34.7)
Clarify doubts	68 (56.2)
**8) When giving bad news, do you always tell the truth about the diagnosis, prognosis and treatment?**	
Give them cautiously, carefully, according to the patient and/or family’s demand	100 (82.6)
Give them all at once	8 (6.6)
Never	13 (10.7)
**9) To whom do you tell the truth to?**	
To the patient and his/her companion at the same time	11 (9.1)
Preferably, first to the family, then to the patient	45 (37.2)
Preferably. first to the patient, then to the family	59 (48.8)
Only to the family	1 (0.8)
Only to the patient	5 (4.1)

^+^Participants can select more than one item in this answer (the sum of percentages will be greater than 100)

Fifty-one (42.2%) respondents frequently or almost always communicate bad news (Q2), and only one (0.8%) stated he never did that task.

Most participants reported using verbal and non-verbal communication when delivering bad news (n = 76, 62.8%, Q3).

Almost two-thirds (61.2%) reported that they seek a private and comfortable place to deliver bad news, while just over a third (37.2%) use an available office, even if it provides less than ideal conditions (Q5). If the patient is bedridden, most of the participants stand near the bed (61.2%), and 38.8% sit on the bed when breaking bad news (Q6, Table 2).

When communicating bad news (n = 110, 90.9%, Q7), most participants reported using clear, understandable language and avoiding technical words, over half (n = 68, 56.2%) clarify patient’s concerns and a third (n = 42, 34.7%) tried to put themselves in the patient’s shoes. Regarding the diagnosis, prognosis, and treatment, most participants (n = 100, 82.6%, Q8) reported that they tell the truth cautiously and carefully according to the patient and/or family’s demand, while only a small part (n = 13, 10.7%) alluded that they never tell the truth.

Nearly half (48.8%) revealed that they communicate the bad news first to the patient and then to the family, while 37.2% communicate them first to the family (Q9, Table 2).

When listening to the patients and their questions, most participants preferred to listen carefully without interruptions (71.1%, Q10) and to spend the necessary time answering questions (66.9%). Although it is a part of a doctor’s role, they may not always feel available or capable of fulfilling this responsibility. Most participants (70%) working in Surgery (n = 16, 76.2%) and Medicine (n = 55, 72.4%) departments were available to answer the patient’s questions and only a third (n = 6, 35.3%) of those working in the Gynecology and Obstetrics were available to undertake this task. In the conversation with the patient, the topics most addressed by the participants are (Q11): understanding what the patients know about their health condition (n = 97, 80.2%) and what concerns them (n = 87, 71.9%). The lowest percentages of participants that explore patient’s concerns are found in the departments of Gynecology and Obstetrics (n = 8, 47.1%) and Surgery (n = 13, 61.9%).

Most participants also reported providing support to the patient and the family, either in the instrumental (n = 83, 68.6%), informational (n = 100, 82.6%), emotional (n = 96, 79.3%) and spiritual (n = 83, 68.6%) aspects (Q14, Table 3). Note that instrumental support is helping in a practical way, through material support, like providing home care to assist in the patient’s daily activities [31].

**Table 3:** Bad news: to whom the physicians provide support when communicating them and what category of support is offered (N = 121)

Question	n (%)	n (%)	n (%)	n (%)
**14) To whom do you offer support?**	Family	Patient	Both	Nobody
14.1) Instrumental	5 (4.1)	21 (17.4)	83 (68.6)	12 (9.9)
14.2) Informational	8 (6.6)	5 (4.1)	100 (82.6)	8 (6.6)
14.3) Emotional	6 (5.0)	11 (9.1)	96 (79.3)	8 (6.6)
14.4) Spiritual	4 (3.3)	14 (11.6)	83 (68.6)	20 (16.5)

There was no association between gender and to whom the participants give their instrumental (p = 0.453), informational (p = 0.518), emotional (p = 0.888), and spiritual (p = 0.926) support. In addition, no statistical differences were found between the time of medical practice and to whom they give their instrumental (p = 0.421), informational (p = 0.366), emotional (p = 0.641), and spiritual (p = 0.959) support.

Regarding the frequency of communicating bad news, significantly more female participants were likely to do it more frequently than male participants (U = 1319; p = 0.003). No significant correlation was found between how often participants deliver bad news and age (r = -0.033, p = 0.717).

Concerning the way participants break bad news (only verbal or both verbal and non-verbal communication), no significant differences were found between: gender (${\chi }^{2}$(1) = 0.126, p = 0.723), time since graduation (U = 1378, p = 0.190) or departments (p = 0.518) of the participants.

No significant differences were found between genders and the place where participants communicate bad news (p = 0.784), or if standing beside the bed when the patient is bedridden (${\chi }^{2}$(1) = 0.163, p = 0.686). Regarding the number of years of practice, no significant differences were found between those who communicate bad news sitting and those who do so standing next to the bed of a bedridden patient (U = 1307, p = 0.054). However, participants who were looking for a private and comfortable place to break bad news were in the profession for significantly more years than those who searched for an available office (median [1st Q; 3rd Q] of 10 [6, 17] versus 7 [3.5, 10.5], U = 1092, p = 0.002).

### Physician’s feelings when giving bad news (Q12 - Q13)

Almost half of the participants (n = 56, 47.9%), reported that they feel sad when communicating bad news, nevertheless 28.2% feel that they have fulfilled their duty (Q12). About fears (Q13), the majority of participants (n = 80, 66.1%) indicated that they fear the patient’s reactions and that they are afraid of shattering the patient’s hope (n = 73, 60.3%, Table 4).

**Table 4:** Feelings of physicians when communicating bad news and training concerning the communication of bad news (N = 121)

Questions	n (%)
^*^ **12) How do you feel about breaking bad news?**	
Relieved	2 (1.7)
Pitying	23 (19.7)
Insecure	3 (2.6)
With a feeling of mission accomplished	33 (28.2)
Sad	56 (47.9)
^+^ **13) What are your fears when delivering bad news?**	
Fear of being blamed	22 (18.2)
Fear of shattering the patient’s hope	73 (60.3)
Fear of death and illness itself	5 (4.1)
Fear of my own emotional reactions	24 (19.8)
Fear of the patient’s reactions	80 (66.1)
**15) How did you learn to deliver bad news?**	
During a specific course	4 (3.3)
During higher education	41 (33.9)
I have not learned it yet	30 (24.8)
By trial-and-error method	14 (11.6)
By observing other colleagues	32 (26.4)
**16) Do you know any instrument that assists in the ability to tell bad news?**	
No	114 (94.2)
Yes	7 (5.8)
**17) How important do you think the incorporation of “How to give bad news” is in higher education?**	
Not important	1 (0.8)
More or less important	2 (1.7)
Important	33 (27.3)
Very important	85 (70.2)
**18) How important do you think the incorporation of** ***“*** **How to give bad news** ***”*** **is in training taking place in hospitals?**	
More or less important	2 (1.7)
Important	23 (19.0)
Very important	96 (79.3)

^*^ 117 answered this question correctly (the other 4 were excluded because they indicated 2 answer options)

^+^ Participants can select more than one item in this answer (the sum of percentages will be greater than 100)

### Learning and knowledge of resources when communicating bad news (Q15-Q18)

Regarding education on the communication of bad news, a third of the participants reported that they learned to communicate bad news during their medical degree, while a fourth stated they did so by observing their medical colleagues in clinical practice. However, there were still participants who indicated that they cannot break bad news (n = 30, 24.8%) (Q15, Table 4). Almost all of the participants (n = 114, 94.2%) did not know any instrument to assist in the development of this skill (Q16, Table 4). No significant differences were found in the distribution of age and years of practice in relation to knowledge or ignorance about instruments that can assist in this task (U = 286, p = 0.226, and U = 285.5, p = 0.547, respectively, Q16).

In addition, most participants (n = 85, 70.2%), believe that is very important to address the communication of bad news during higher education, and 27.3% consider it important (Q17). Concerning the address of this theme in training, 79.3% of the participants see that as very important and 19% as important (Q18, Table 4).

## Discussion

Following the recent implementation of palliative care in Mozambique, this study explores the communication strategies used by physicians involved in providing palliative care at one hospital. Overall, the results demonstrated that most participants understand the concept of bad news, delivering it occasionally or frequently during their daily practice. However, a large part considered their ability to communicate bad news only as acceptable, which may be related to the unfamiliarity with the tools that can assist them in this challenging task [21]. Pereira et al. 2017 work demonstrated that a significant part of the physicians considered their communication skills as reasonable and had never received any training to help in this task, but agreed that the implementation of the P-A-C-I-E-N-T-E mnemonic would be very useful to increase their experience [5]. Our study identified similar gaps in physicians communicating bad news training.

Although more than half of the participants of this study used verbal and non-verbal language when communicating bad news to their patients, it was still found to be a lower result when compared to the study of Pereira et al. 2017. In this one, medical doctors with more experience used verbal and non-verbal communication, as greater clinical experience enables interaction with patients and families and their emotions [5, 18]. The use of non-verbal communication appears to support patients in expressing their spiritual needs, which improves the patient-physician relationship [32]. The integration of palliative care into the National Health System in Mozambique may allow the structuring of palliative care at the MCH and improve the training of healthcare professionals, increasing the use of non-verbal and verbal language, which can facilitate the empathetic communication between patient and physician [33].

An interesting result found is the significantly higher frequency of women that communicate bad news, unlike in other studies conducted in Iran and Brazil where the diagnosis is most commonly delivered by older and more experienced male physicians [18, 20]. The difference observed between genders may be related to cultural issues such as the role of women in health in Mozambique regarding contact with patients, since they seem to have better social abilities than men, which together with the higher educational level may contribute to an increase of their communication skills. It was already reported that female physicians are more empathic, positive, and affective when communicating with patients [34].

Regarding the approaches used by doctors when communicating bad news, besides looking for a private and comfortable place, most referred to do it clearly, carefully, and with understandable language. Moreover, a significant part would set aside a period to clarify doubts, according to the demands of the patient and/or family, and listen to the patient carefully, without interruption. The physicians also reported that they were most focused on listening to what patients already knew about their condition and/or concerns. Additionally, the majority of the research participants told the truth first to the patient and then to the family. A study developed in Brazil and another in China [21, 35] observed that most physicians told the truth first to the family and then to the patient. These differences may be related to cultural traits [36] and/or unpreparedness to deal with the patients’ emotions [18].

Participants of this study demonstrated feelings of insecurity and fears regarding the communication of bad news. A large percentage felt sad, and afraid of being blamed for the impossibility of curing patients, in addition to fearing patients’ reactions, similar to the results obtained in the research developed in Brazil [21]. These difficulties in dealing with their own feelings and those of patients can activate physicians’ psychological defense mechanisms, further hindering communication [37]. Moreover, in a study conducted in a Chinese hospital about physician’s concerns when informing of a cancer diagnosis or prognosis, the main reasons found to hide bad news were the fear that most patients could not cope with them, fear of conflict with the patient’s family, and of get caught in a difficult situation where they need to choose between respecting the patient’s “right to know” and respecting the family’s opinion in what concerns to protect the patient [35]. In a study conducted at the Iman Cancer Institute, in Iran, the fear of patients’ emotional reactions was the most important factor for physicians’ refusal to communicate the diagnosis, as they feel unable to deal with patients’ emotions [18]. These reports reinforce the need for training professionals to deal with the patient’s and family’s emotions and with their own feelings. Recently, a study with dermatology residents demonstrated that simulating the delivery of bad news can be very useful in dealing with stress and improving the attitude and communication skills, especially for professionals with less experience [38]. Also, a study from Botswana revealed that role-play training, with an approach to SPIKES mnemonic, can help medical students improve their skills and confidence to break bad news [39].

Worryingly, most of the participants of this study indicated that they had no training related to communication of bad news and highlighted the importance of the inclusion of this area in the academic curriculum, and in continuous training. In fact, some participants reported that they learned to communicate bad news by observing other professionals, which raises the question of how prepared are the doctors who act as models [40].

With this study, it is highlighted the need for doctors in the palliative care area to develop communication skills, self-assessment capacity and a sense of reality, as these deficiencies generate issues in terms of context, culture, language, as well as lack of empathy [19–21, 32, 36].

Moreover, conducting research that explores the perspectives and experiences of patients, their family members, and doctors in the context of delivering bad news, could provide valuable insights into the communication process and the support offered by healthcare professionals to patients, their families, and caregivers [21, 41].

### Limitations

This is an exploratory study and thus further studies are necessary to be conducted to confirm and extend the present findings. Nevertheless, some limitations can be pointed out. The first is that the results are based on data obtained from only one hospital in Mozambique, so they may not be generalized to the whole country. The second is that the instrument was a self-filling questionnaire, which originated some missing data (ex. question Q12). Third, the expert committee evaluating the pertinence and relevance of the survey are the authors of this article. Fourth, as palliative care is an area that is beginning to be implemented in Mozambique and physicians are not used to participating in research regarding their conduct, it may have an information bias related to the intention of some participants to state what they thought was more adequate. They had difficulties informing the truth to the patient, opting for paternalistic behavior to protect him from pain and suffering, as well as avoiding their own anxiety in the process of breaking bad news.

### Recommendations

Given the above, it is considered necessary the expansion of this line of research in palliative care, so that the results help in the implementation and improvement of patient care, including research involving patients and family members to verify the effectiveness of the communication procedure. It is also pertinent and relevant to publicize the interest and incorporation of the topic of communication in the context of palliative care in undergraduate training, as well as in hospital practice and training. Furthermore, it is mandatory the use of appropriate protocols for the reality of palliative care in Mozambique, which should include all those involved in the process, with the aim to improve the attitudes of physicians towards patients and families when the treatment becomes palliative, instead of cure.

## Data Availability

The data generated and analyzed during the current study are not publicly available due to confidentiality reasons but are available from the corresponding author upon reasonable request.

## List of abbreviations

MCH: Maputo Central Hospital
SPIKES: Setting-Perception-Invitation-Knowledge-Emotions-Strategy
WHO: World Health Organization
